# Comparisons of the in-the-bag stabilities of single-piece and three-piece intraocular lenses for age-related cataract patients: a randomized controlled trial

**DOI:** 10.1186/s12886-016-0283-4

**Published:** 2016-07-08

**Authors:** Xiaojian Zhong, Erping Long, Wan Chen, Wu Xiang, Zhaochuan Liu, Hui Chen, Jingjing Chen, Zhuoling Lin, Haotian Lin, Weirong Chen

**Affiliations:** State Key Laboratory of Ophthalmology, Zhongshan Ophthalmic Center, Sun Yat-sen University, Guangzhou, Guangdong 510060 China

**Keywords:** Cataract surgery, In-the-bag stabilities, Single-piece intraocular lenses, Three-piece intraocular lenses

## Abstract

**Background:**

To compare the in-the-bag stability and visual function of single-piece intraocular lenses (IOLs) and three-piece IOLs.

**Methods:**

A total of 65 patients with age-related cataracts (80 eyes) were enrolled and randomly assigned to receive in-the-bag implantation of either a single-piece IOL (40 eyes) or a three-piece IOL (40 eyes). Follow-up visits were conducted at 1 week, 1 month and 3 months postoperatively. Visual acuity, refraction and total aberration were examined. IOL position stability (including axial movement, decentration and tilt) was measured using a Scheimpflug imaging system.

**Results:**

At the 3-month follow-up visit, single-piece IOLs did not exhibit significant axial movement (0.07 ± 0.30 mm, *p* = 0.13) compared with their axial position at 1 week postoperatively, whereas three-piece IOLs displayed forward axial movement of −0.22 ± 0.23 mm (*p* < 0.0001). The mean manifest spherical equivalence (SE) of eyes with single-piece IOL was 0.15 ± 0.18D, whereas in eyes with three-piece IOLs, the mean manifest SE was −0.34 ± 0.15D (*p* < 0.001). There was no statistically significant difference in IOL decentration, tilt, uncorrected visual acuity, best-corrected visual acuity or total spherical aberration between the two groups.

**Conclusions:**

Three months after implantation, single-piece IOLs exhibit better axial stability and more stable refractive outcome than three-piece IOLs, but both IOLs perform equally well in terms of decentration, tilt, visual acuity and total aberration.

**Trial registration:**

ClinicalTrial.gov, NCT02609997, 11/18/2015, retrospectively registered.

**Electronic supplementary material:**

The online version of this article (doi:10.1186/s12886-016-0283-4) contains supplementary material, which is available to authorized users.

## Background

Rapid advances in cataract surgery techniques and intraocular lens (IOL) technology have enabled the transition of cataract surgery from blindness relief to refractive correction [1]. An ideal IOL is the critical component to achieve the refractive target of cataract surgery. Biocompatibility, rate of posterior capsule opacification (PCO) and visual quality have all been suggested as critical characteristics of an ideal IOL and widely investigated. Stability of IOL position was also recently proposed as a critical factor due to its close correlation with postoperative visual function. An IOL forward movement of 0.29 mm along the visual axis has been associated with a myopic shift of −0.4D [2]. Meanwhile, Wang and colleagues recently reported that 0.5-mm decentration of an aspheric IOL could eliminate its aberration-correcting effect [3]. Moreover, poor stability could even lead to IOL exchange as well as additional surgery, which both surgeons and patients wish to avoid.

As the supporting element, haptics are crucial to maintaining the position of the IOL. Various haptic designs have been compared in terms of the position stability of IOLs. Haptic designs of single-piece versus three-piece IOLs are often compared because these are the most commonly used types. Single-piece IOLs have soft and broader haptics that are manufactured from the same material as the optic, usually hydrophobic or hydrophilic acrylic, whereas three-piece IOLs have rigid haptics that are composed of poly methyl methacrylate (PMMA). Clinical studies comparing these haptic designs have yielded conflicting results regarding their position stability in the capsular bag, the most-recommended site for IOL fixation in an uneventful cataract surgery.

Most previous studies have measured the IOL position based on Purkinje reflections [4]. However, this measurement is time-consuming, and patients are reluctant to cooperate during image acquisition [5]. In addition, Purkinje measurement does not detect anterior chamber depth (ACD) and thus fails to reveal the IOL position along the axis. By contrast, clinical Scheimpflug systems based on rotating Scheimpflug imaging can acquire enough 3-dimensioinal data points within a reasonably short period, usually seconds, and are one of the best methods to estimate IOL position [5].

To better compare the intracapsular stability of single-piece and three-piece IOLs, we measured IOL positions using rotating Scheimpflug imaging systems and tested the visual quality of patients implanted with these IOLs.

## Methods

The protocol for this trial is available as supplementary information; see Additional file 1.

### Patients

A total of 65 patients with age-related cataracts (80 eyes) were enrolled between December 2012 and December 2013 from Zhongshan Ophthalmic Center (ZOC) [6], which is China’s largest eye hospital and is located in Guangzhou city, South China.

### Inclusion and exclusion criteria

#### The inclusion criteria

- Patients with a diagnosis of bilateral age-related cataracts
- Age between 60 and 85 years.

#### The exclusion criteria

- Diagnosis of vision-impairing diseases other than cataracts, severe refractive error (preoperative spherical equivalent of either eye > −6.00D or +5.00D);
- History of ocular trauma; past refractive surgery or other ophthalmic surgery;
- Capsular or zonular disorders that might affect the post-operative centration of IOLs, e.g., pseudo-exfoliation syndrome or Marfan syndrome;
- Surgical complications including severe hyphema, iris injury, repeated IOL implantation during surgery, failure to achieve in-the-bag IOL implantation and corneal sutures.

### Randomization and masking

**Fig. 1:**
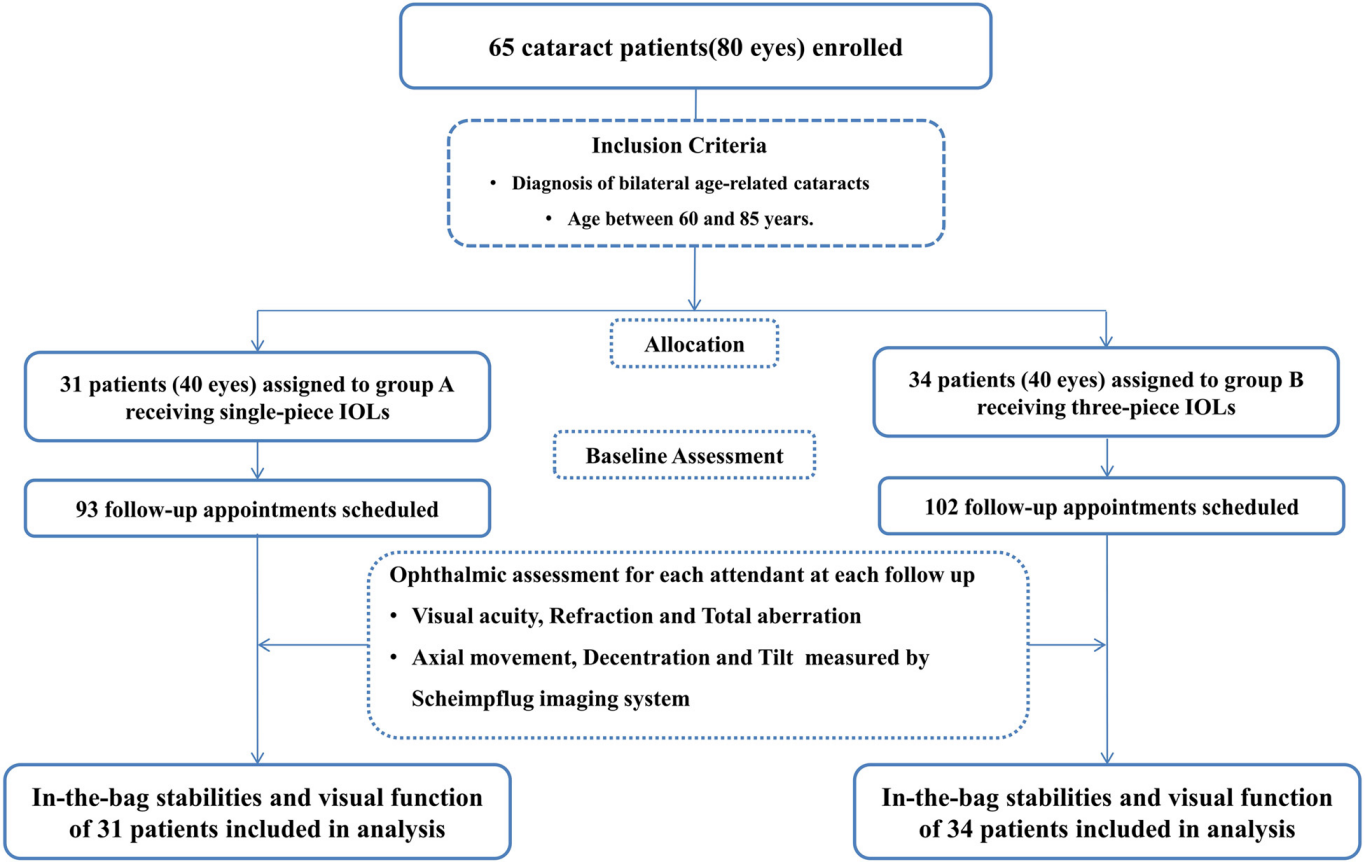
Flow chart of the patient selection and follow-up protocols. (Note: IOL = intraocular lens)

Participants were assigned by simple randomization (1:1) to either Group A, receiving single-piece IOLs (ZCB00, Abbot Medical Optics, Illinois, USA) or Group B receiving three-piece IOLs (ZA9003, Abbot Medical Optics, Illinois, USA) [7]. The randomization codes were generated using a random number generating program (Random number generator tools, version 1.4, Duote Co., Wuhu, China). Written allocation assignments were sealed in individual opaque envelopes marked only with study identification numbers. Patients were blinded to the study design and the actual IOL type they received (Fig. 1). Regular ocular examinations and analyses were performed by investigators and clinical staff, both masked to group allocation. Study personnel in charge of randomization and the ophthalmic surgeons could not be masked because the intervention required overt participation.

### Intraoperative and postoperative procedures

All patients underwent standard phacoemulsification cataract extraction performed by a single experienced cataract surgeon (WRC). Topical anesthesia consisting of a single drop of 0.5 % proparacaine (Alcaine, Alcon Laboratories, Inc, Texas, USA), was administered three times at 5-min intervals prior to surgery. A 3.2-mm temporal corneal incision was followed by 5.5-mm capsulorhexis, hydrodissection and phacoemulsification of the nucleus, irrigation/aspiration of the remaining cortex, in-the-bag implantation of the IOL and final hydration of the incision. The target refraction was set at emmetropia for all patients.

Postoperative topical therapy included 0.3 % tobramycin and 0.1 % dexamethasone eye drops (Tobradex, Alcon Laboratories, Inc, Texas, USA) four times per day and 0.3 % tobramycin and 0.1 % dexamethasone eye ointment (Tobradex, Alcon Laboratories, Inc, Texas, USA) every night for one month.

### Follow-up protocol and assessment methods

**Fig. 2:**
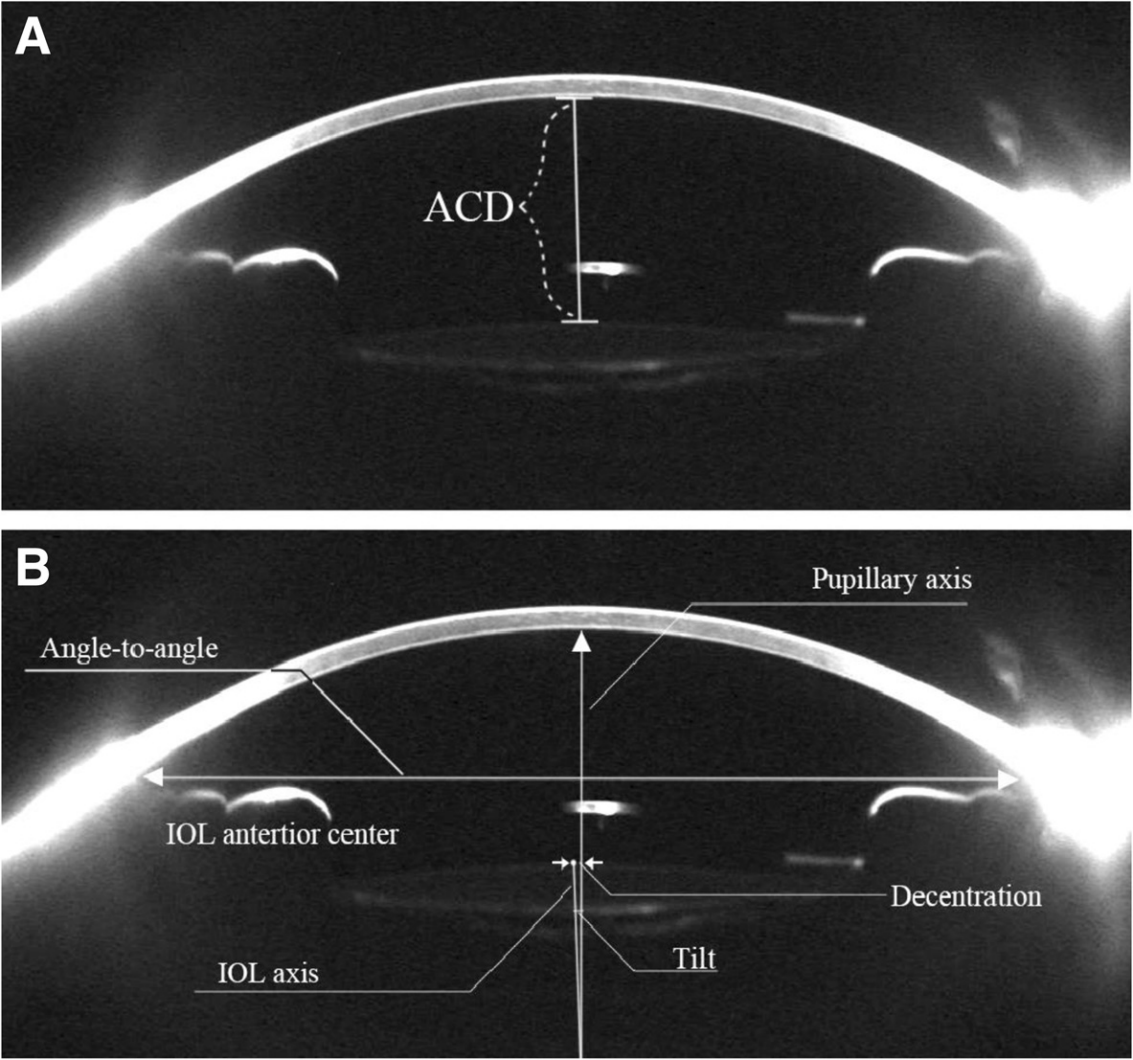
Scheimpflug image of the horizontal cross-section of the anterior segment after IOL implantation. Panel **a**: The central anterior chamber depth (ACD) was measured from the central corneal posterior endothelium to the IOL anterior surface. Panel **b**: Schematic drawing of the decentration and tilt measurement. Decentration was measured from the center of the IOL anterior surface to the pupillary axis. Tilt was measured as the angle between the IOL axis and the pupillary axis

Follow-up examinations were scheduled 1 week, 1 month and 3 months postoperatively. A comprehensive ophthalmic examination was performed during each visit. Visual function was examined before IOL position. When evaluating visual function, a Snellen chart was used to assess visual acuity, and the outcome was converted to logMAR [8]. Manifest refraction was performed and further used to determine the spherical equivalent (SE) and best-corrected visual acuity. Total aberration was measured using an iTrace aberrometer (Tracey Technologies, Inc, Texas, USA) under dark lighting conditions. Pupil dilation was then induced by instilling 0.5 % tropicamide eyedrops. When the pupil was sufficiently dilated, IOL position was measured using Pentacam (OCULUS Optikgeräte GmbH, Germany) as shown in Fig. 2. The Scheimpflug image of the horizontal cross section of the target eye was selected for measurement. Central ACD was measured as the distance between the central corneal posterior endothelium and the anterior surface of IOL (Fig. 2 Panel a). The subtraction in ACD between two visits indicated forward or backward axial movement of the IOL. Decentration was measured from the center of IOL anterior surface to the pupillary axis, which was perpendicular to the line between the two anterior chamber angles and through the midpoint of the line. Tilt was measured as the angle between the IOL axis and the pupillary axis (Fig. 2 Panel b). All measurements were performed by experienced ophthalmic technicians who were blinded to the aim of the study as well as the patient’s IOL type.

### Statistical analysis

The sample size calculation was based on power analysis. Power analysis adopts a hypothesis-testing method to determine the sample size according to several parameters, which include the pre-specified significance level, desired power level and expected effect size. Assuming a two-tailed alpha of 0.05, a probability of 0.2 for beta error (80 % power) and the reported results of similar research [9] as our reference rate, 40 participants per group were required.

Demographic and clinical information were recorded at baseline. Statistical analysis was performed using the Statistical Package for the Social Sciences (SPSS ver. 17.0.1, SPSS Inc., Chicago, IL, USA). The normality of the data distribution was assessed using the Kolmogorov-Smirnov test. An unpaired *t* test was performed to compare means of continuous variables that exhibited normal distributions (axial movement, decentration, tilt, visual acuity, refraction and total aberration) between the two groups. Continuous variables without a normal distribution were compared using the Mann–Whitney U test. Categorical variables such as sex were compared using Fisher’s exact probability test. Because of the nested structure of the eyes, we additionally applied a generalized estimating equation with a working covariance matrix of unstructured correlations to analyze repeated measures data. All statistical tests were two-tailed, and a p-value below 0.05 was considered statistically significant. The results are presented as the mean ± standard deviation (SD).

## Results

Sixty-five patients (80 eyes) with age-related cataracts completed the 3-month follow-up study. After enrollment, the patients were randomly allocated into two groups. The 31 patients (40 eyes) in Group A received single-piece IOLs, whereas the 34 patients (40 eyes) in Group B and received three-piece IOLs. The baseline data for the study subjects are summarized in Table 1. There was no statistically significant difference between the two groups with regard to age (*p* = 0.32), sex (*p* > 0.99), axial length (*p* = 0.64) or ACD (*p* = 0.42).

**Table 1:** Baseline characteristics of the participants in the present study comparing the in-the-bag stabilities of single-piece and three-piece intraocular lenses

	Group A (Single piece IOL) Mean ± SD	Group B (Three-piece IOL) Mean ± SD
Age	73.3 ± 9.3 years	71.4 ± 7.7 years
	(Range 60–85 years)	(Range 61–83 years)
Axial Length	23.42 ± 1.35 mm	23.53 ± 1.18 mm
ACD	2.67 ± 0.45 mm	2.75 ± 0.49 mm
Gender	n (%)	n (%)
Male	14 (45.2)	15 (44.1)
Female	17 (54.8)	19 (55.9)

None of baseline characteristics differed significantly between the two groups at the 0.05 level. Notes: *IOL* intraocular lens, *ACD* anterior chamber depth, *SD* standard deviation

The axial movement, decentration and tilt of the two IOLs are presented in Table 2. At the 3-month visit, the single-piece IOLs did not exhibit forward or backward movement with regards to the axial position compared to 1 week (0.07 ± 0.30 mm, *p* = 0.13). By contrast, the three-piece IOLs displayed forward axial movement (−0.22 ± 0.23 mm, *p* < 0.0001). The single-piece IOLs displayed 221 ± 167 μm decentration, whereas the three-piece IOLs exhibited 198 ± 165 μm decentration. The difference between the two IOLs was not statistically significant (*p* = 0.56). The tilt measurements for the single-piece and three-piece IOLs were 1.06 ± 0.49° and 1.01 ± 0.45°, respectively (*p* = 0.66).

**Table 2:** In-the-bag stability of single-piece and three-piece IOLs at 3 months

	Group A (single-piece IOL) Mean ± SD	Group B (three-piece IOL) Mean ± SD
Axial movement	0.07 ± 0.30 mm	−0.22 ± 0.23 mm*
Decentration	221 ± 167 μm	198 ± 165 μm
Tilt	1.06 ± 0.49°	1.01 ± 0.45°

The axial movement, decentration and tilt of the two IOLs are presented in Table 2. At the 3-month postoperative visit, the 3-piece IOLs displayed forward axial movement (−0.22 ± 0.23 mm, *p* < 0.0001) (*p < 0.05). The single-piece IOLs displayed 221 ± 167 μm decentration, whereas the 3-piece IOLs exhibited 198 ± 165 μm decentration. Notes: *IOL* intraocular lens, *SD* standard deviation

The visual performance of the two IOLs is presented in Table 3. The mean uncorrected visual acuity was 0.04 ± 0.06 logMAR in the single-piece IOL group and 0.04 ± 0.08 logMAR in the three-piece IOL group (*p* = 1.00). The mean best-corrected visual acuity was 0.02 ± 0.09 logMAR in the single-piece IOL group and 0.03 ± 0.08 logMAR in the three-piece IOL group (*p* = 1.00). Manifest SE at 3 months postoperatively was 0.15 ± 0.18 D in the single-piece IOL group and −0.34 ± 0.15 D in the three-piece IOL group (*p* < 0.001). The mean total aberration was 0.03 ± 0.06 μm in the single-piece IOL group and 0.02 ± 0.07 μm in the three-piece IOL group (*p* = 0.94).

**Table 3:** Visual function of eyes with single-piece and three-piece IOLs at 3 months

	Group A (single-piece IOL) Mean ± SD	Group B (three-piece IOL) Mean ± SD	*p*-value
UCVA	0.04 ± 0.06 (logMAR)	0.04 ± 0.08 (logMAR)	1.00
BCVA	0.02 ± 0.09 (logMAR)	0.03 ± 0.08 (logMAR)	1.00
SE	0.15 ± 0.18 D	−0.34 ± 0.15 D	<0.001*
Total aberration	0.03 ± 0.06 μm	0.02 ± 0.07 μm	0.94

The visual performance of the two IOLs is presented in Table 3. Manifest SE at 3 months postoperatively was 0.15 ± 0.18 D in the single-piece IOL group and −0.34 ± 0.15 D in the three-piece IOL group (*p* < 0.001)(*p < 0.05) . Notes: *IOL* intraocular lens, *SD* standard deviation, *UCVA* uncorrected visual acuity, *BCVA* best-corrected visual acuity, *SE* spherical equivalent

## Discussion

IOL position stability is closely related to postoperative visual function. Various factors affect position stability. Our study compared the position stability of single-piece and three-piece aspheric IOLs in a clinical scenario. The results revealed that single-piece IOLs moved less than three-piece IOLs along the visual axis and therefore had greater axial stability. The forward movement of three-piece IOLs caused a slight myopic shift, whereas the refractive status of single-piece IOLs remained stable. This result is consistent with previous studies [9, 10].

In a laboratory study of the biomechanical properties of IOLs with different haptic designs [11], Lane et al. demonstrated that the optic of a single-piece IOL exhibits significantly less axial movement than that of three-piece IOLs during haptic compression. After implantation in the capsular bag, the haptics undergo compression, particularly in the case of capsular contraction, and soft haptics exert less force on the optic, resulting in less axial movement of the IOL. Axial movement beyond a certain extent will lead to “refractive surprises” and unsatisfactory visual function. In the worst scenario, a refractive surprise would require IOL exchange [12]. Therefore, single-piece IOLs might be more appropriate for implantation in the capsular bag due to better position stability.

Decentration and tilt of single-piece and three-piece spherical IOLs have been investigated in multiple studies [13]. Our observations of aspheric IOLs are consistent with these studies and indicate that the decentration and tilt of both types of IOLs are not clinically significant. Aspheric IOLs are unique in that they pose greater demands on centration. McKelvie et al. investigated the association of IOL decentration and tilt with aberration and suggested decentration greater than 0.5 mm and tilt greater than 4° are clinically significant for aspheric IOLs [14]. In our study, the decentration and tilt were within these limits in both groups, and the total aberration in both groups was close to zero, consistent with McKelvie’s results.

Previous comparisons of the position stability of different IOLs have primarily used Purkinje imaging to measure IOL position in the eye. For this method, a point-light source is fixed at a certain distance in front of the eye. The patient is asked to stare at the light source, and reflections of the light source form at the front cornea, back of the cornea and anterior surface of IOL. The image of these reflections is recorded, and the distances between any two reflections are measured and used to calculate the decentration and tilt of the IOL [15]. The Purkinje method is the gold standard for measuring IOL decentration and tilt [16]. However, the use of the Purkinje method among patients with age-related cataracts is hindered by low compliance by elderly subjects. Keeping the eyelids wide open and staring at a light source for a certain time can be challenging for these patients. Furthermore, the Purkinje method cannot measure the axial position of the IOL. Our study used a rotating Scheimpflug imaging system (Pentacam) to measure the position of the IOL in the eye. Rosales et al. determined that Purkinje and Scheimpflug have comparable accuracy and repeatability [17]. An advantage of the Scheimpflug imaging system is that the image acquisition time is 3 s or less; thus, most patients are able to cooperate well. Few clinical studies have employed the Scheimpflug imaging system, and further studies are needed to confirm its diagnostic value.

The results and interpretation of the current study must be understood within the context of its strengths and limitations. Few clinical studies have used a rotating Scheimpflug imaging device to compare stability in the capsular bag between single-piece and three-piece IOLs. The process of image acquisition and data generation is independent of the operator; the measurement is therefore objective, and the results provide additional clinical evidence. This is the major strength of the present study. However, our study features several limitations as well. First, patients were followed up only for three months. This short follow-up period is not fully adequate to determine if single-piece IOLs or three-piece IOLs are more appropriate for implantation in the capsular bag. Second, the sample size of the present study was insufficient to detect differences in decentration and tilt. Third, fellow-eye comparison was not performed due to ethical considerations. Despite these limitations, the results of this study are useful given the lack of clinical investigations of aspheric IOLs with different haptic designs. Further studies with longer follow-up periods, larger sample sizes and more rigorous designs are needed.

## Conclusions

In summary, the current study compared the position stability of single-piece and three-piece aspheric IOLs using a rotating Scheimpflug imaging system. The axial movement of single-piece IOLs was significantly less than that of three-piece IOLs. The forward movement of the three-piece IOL was associated with myopic shift. The aspheric IOLs were similar in the amount of decentration, tilt, visual acuity and total aberration. Further studies are needed to evaluate the long-term stability and visual function of these IOL designs.

## Abbreviations

ACD, anterior chamber depth; IOL, intraocular lenses; PCO, posterior capsule opacification; PMMA, poly methyl methacrylate; SD, standard deviation; SE, spherical equivalent; ZOC, Zhongshan Ophthalmic Center

## Additional file

Additional file 1: Study protocol for this trial. The study protocol file contains six sections: background and purpose, patient recruitment and enrollment, surgical protocols, follow-up and evaluation of surgical outcomes, statistical analyses and reference. (DOCX 45 kb)
