# Supplementary material for: Comparisons of the in-the-bag stabilities of single-piece and three-piece intraocular lenses for age-related cataract patients: a randomized controlled trial
Source: BMC Ophthalmol. 2016 Jul 8;16:100. doi: 10.1186/s12886-016-0283-4 (PMC4939005; doi:10.1186/s12886-016-0283-4)
Supplement: Additional file 1: — Study protocol for this trial. The study protocol file contains six sections: background and purpose, patient recruitment and enrollment, surgical protocols, follow-up and evaluation of surgical outcomes, statistical analyses and reference. (DOCX 45 kb) [file 12886_2016_283_MOESM1_ESM.docx]

**Supplementary information**

**Comparisons of the in-the-bag stabilities of single-piece and three-piece intraocular lenses for age-related cataract patients: A randomized controlled trial**

**Co-authors:** Xiaojian Zhong^¶^, Erping Long^¶^, Wan Chen, Wu Xiang, Zhaochuan Liu, Hui Chen, Jingjing Chen, Zhuoling Lin, Haotian Lin^§^, Weirong Chen^§^

**Institution:** State Key Laboratory of Ophthalmology, Zhongshan Ophthalmic Center, Sun Yat-sen University, Guangzhou, Guangdong, 510060, China

^¶^These authors contributed equally to this work.

^§^Co-corresponding authors:

Professor Haotian Lin, M.D., Ph.D., E-mail:haot.lin@hotmail.com; Professor Weirong Chen, M.D., Email: [chenwr_q@aliyun.com](mailto:chenwr_q@aliyun.com); Telephone: +86-20-87330493; Fax: +86-20-87333271; Address: Xian Lie South Road 54#, Guangzhou, China, 510060

**- Additional file 1.**

**- Study protocol for this trial.**

**Comparisons of the in-the-bag stabilities of single-piece and three-piece intraocular lenses for age-related cataract patients: A randomized controlled trial**

**Study Protocol**

**Table of Contents**

1. Background and Purpose………………………………………………………………...3

2. Patient Recruitment and Enrollment …………………………………………………….7

3. Surgical Protocols ……………………....……………………………………………….9

4. Follow-up and evaluation of surgical outcomes………………………………………...10

5. Statistical Analyses……………………………………………………………………...11

6. References……………………………………………………………………………….12

**Chapter 1**

**Background and Purpose**

**1.1 Objectives**

This study is a randomized clinical study with the following objectives:

- To compare the IOL position stability (including axial movement, decentration and tilt) between single-piece and three-piece intraocular lenses.
- To compare the visual function (visual acuity, refraction and total aberration) between single-piece and three-piece intraocular lenses.

**1.2 Rationale of the Study**

The study is conducted for the following reasons:

- Rapid advances of cataract surgery techniques and intraocular lens (IOL) technology have enabled the transition of cataract surgery from blindness relief to refractive correction. An ideal IOL is the critical component to achieve the refractive target of cataract surgery. Biocompatibility, rate of posterior capsule opacification (PCO) and visual quality have all been suggested as the critical factors of an ideal IOL and widely investigated. Recently, stability of IOL position has also been suggested as one of those critical factors due to its close correlation with postoperative visual function. Data suggests that IOL forward movement of 0.29 mm along the visual axis is associated with -0.4D myopic shift. Wang and colleagues recently reported that 0.5mm decentration of an aspheric IOL could eliminate its aberration-correcting effect. Poor stability could even lead to IOL exchange, an additional surgery that put both surgeons and patients in pain.
- As the supporting element of an IOL, the haptics are crucial to keep the IOL in place. Various haptic designs are being compared in terms of position stability of IOLs. Haptic designs of single-piece versus 3-piece are often compared because they are currently the most commonly used types. Single-piece IOLs have soft and broader haptics which are made of the same material as the optic, usually hydrophobic or hydrophilic acrylic, whereas 3-piece IOLs have rigid haptics which are made of poly methyl methacrylate (PMMA). Clinical studies comparing these haptic designs have yielded controversial results regarding their position stability in the capsular bag, which is the most recommended site for IOL fixation in an uneventful cataract surgery. Our investigation may provide new insights into the optimal surgical timing for patients of congenital cataracts.

**1.3 Summary of Study Protocol**

**Major eligibility criteria:**

- Patients with a diagnosis of bilateral age-related cataracts
- Age between 60 and 85 years.
- Patients without vision-impairing diseases other than cataracts, severe refractive error (preoperative spherical equivalent of either eye >-6.00D or +5.00D);
- Patients without history of ocular trauma; past refractive surgery or other ophthalmic surgery;
- Patients without capsular or zonular disorders that might affect the post-operative centration of IOLs,
- Patients without surgical complications including severe hyphema, iris injury, repeated IOL implantation during surgery, failure to achieve in-the-bag IOL implantation and corneal sutures.
- Who could not complete the follow-up was excluded.

**Treatment groups:** All patients underwent standard phacoemulsification cataract extraction performed by the single experienced cataract surgeon (WRC). Topical anesthesia consisting of a single drop of 0.5% proparacaine (Alcaine, Alcon Laboratories, Inc, Texas, USA), was administered three times at 5-minute intervals prior to surgery. A 3.2-mm temporal corneal incision was followed by 5.5-mm capsulorhexis, hydrodissection and phacoemulsification of the nucleus, irrigation/aspiration of the remaining cortex, in-the-bag implantation of the IOL and final hydration of the incision. The target refraction was set at emmetropia for all patients. Postoperative topical therapy included 0.3% tobramycin and 0.1% dexamethasone eye drops (Tobradex, Alcon Laboratories, Inc, Texas, USA) four times per day and 0.3% tobramycin and 0.1% dexamethasone eye ointment (Tobradex, Alcon Laboratories, Inc, Texas, USA) every night for one month.

**Examination Schedule:** A comprehensive ophthalmic examination was performed during each visit. Visual function was examined before IOL position. When evaluating visual function, a Snellen chart was used to assess visual acuity, and the outcome was converted to logMAR. Manifest refraction was performed and further used to determine the spherical equivalent (SE) and best-corrected visual acuity. Total aberration was measured using an iTrace aberrometer (Tracey Technologies Inc, Texas, and USA) under dark lighting conditions. Pupil dilation was then induced by instilling 0.5% tropicamide eyedrops. When the pupil was sufficiently dilated, IOL position was measured using Pencatam (OCULUS Optikgeräte GmbH, Germany). The Scheimpflug image of the horizontal cross section of the target eye was selected for measurement. Central ACD was measured as the distance between the central corneal posterior endothelium to the anterior surface of IOL. The subtraction in ACD between two visits indicated forward or backward axial movement of the IOL. Decentration was measured from the center of IOL anterior surface to the pupillary axis, which was perpendicular to the line between the two anterior chamber angles and through the midpoint of the line. Tilt was measured as the angle between the IOL axis and the pupillary axis.

**Primary Outcome**: IOL position stability and visual functions of the postoperative cataract patients.

**Chapter 2**

**Patient Recruitment and Enrollment**

**2.1 Participant Recruitment**

A total of 65 patients with age-related cataracts (80 eyes) were enrolled between December 2012 and December 2013 from Zhongshan Ophthalmic Center (ZOC), which is China’s largest eye hospital and is located in Guangzhou city, South China.

**2.2 Inclusion and Exclusion Criteria**

*The inclusion criteria:*

- Patients with a diagnosis of bilateral age-related cataracts
- Age between 60 and 85 years.

*The exclusion criteria:*

- Diagnosis of vision-impairing diseases other than cataracts, severe refractive error (preoperative spherical equivalent of either eye >-6.00D or +5.00D);
- History of ocular trauma; past refractive surgery or other ophthalmic surgery;
- Capsular or zonular disorders that might affect the post-operative centration of IOLs, e.g., pseudo-exfoliation syndrome or Marfan syndrome;
- Surgical complications including severe hyphema, iris injury, repeated IOL implantation during surgery, failure to achieve in-the-bag IOL implantation and corneal sutures.

**2.3 Examination Procedures**

**Ophthalmic examination will assess:**

1. Visual acuity measured by Snellen chart
2. Refraction
3. Total aberration
4. Axial movement
5. Decentration
6. Tilt

**Chapter 3**

**Surgical Protocols**

**3.1 Treatment Groups**

Patients will be assigned to one of two treatments:

1. Group A received cataract surgery with single-piece intraocular lens implantation.
2. Group B received cataract surgery with three-piece intraocular lens implantation.

**3.2 Surgical Protocols**

All patients underwent standard phacoemulsification cataract extraction performed by the single experienced cataract surgeon (WRC). Topical anesthesia consisting of a single drop of 0.5% proparacaine (Alcaine, Alcon Laboratories, Inc, Texas, USA), was administered three times at 5-minute intervals prior to surgery. A 3.2-mm temporal corneal incision was followed by 5.5-mm capsulorhexis, hydrodissection and phacoemulsification of the nucleus, irrigation/aspiration of the remaining cortex, in-the-bag implantation of the IOL and final hydration of the incision. The target refraction was set at emmetropia for all patients.

**3.3 Postoperative Medical Therapy**

Postoperative topical therapy included 0.3% tobramycin and 0.1% dexamethasone eye drops (Tobradex, Alcon Laboratories, Inc, Texas, USA) four times per day and 0.3% tobramycin and 0.1% dexamethasone eye ointment (Tobradex, Alcon Laboratories, Inc, Texas, USA) every night for one month.

**Chapter 4**

**Follow-up and evaluation of surgical outcomes**

**4.1 Follow-up Examination Schedule**

All patients returned for follow up visits at 1 week, 1 month and 3 month after surgery.

**4.2 Follow-up Examination Procedures**

Follow-up examinations were scheduled 1 week, 1 month and 3 months postoperatively. A comprehensive ophthalmic examination was performed during each visit. Visual function was examined before IOL position. When evaluating visual function, a Snellen chart was used to assess visual acuity, and the outcome was converted to logMAR. Manifest refraction was performed and further used to determine the spherical equivalent (SE) and best-corrected visual acuity. Total aberration was measured using an iTrace aberrometer (Tracey Technologies Inc, Texas, and USA) under dark lighting conditions. Pupil dilation was then induced by instilling 0.5% tropicamide eyedrops. When the pupil was sufficiently dilated, IOL position was measured using Pencatam (OCULUS Optikgeräte GmbH, Germany). The Scheimpflug image of the horizontal cross section of the target eye was selected for measurement. Central ACD was measured as the distance between the central corneal posterior endothelium to the anterior surface of IOL. The subtraction in ACD between two visits indicated forward or backward axial movement of the IOL. Decentration was measured from the center of IOL anterior surface to the pupillary axis, which was perpendicular to the line between the two anterior chamber angles and through the midpoint of the line. Tilt was measured as the angle between the IOL axis and the pupillary axis.

**Chapter 5**

**Statistical Analyses**

The sample size calculation was based on power analysis. Power analysis adopts a hypothesis-testing method to determine the sample size according to several parameters, which include the pre-specified significance level, desired power level and expected effect size. Assuming a two-tailed alpha of 0.05, a probability of 0.2 for beta error (80% power) and the reported result of previous similar research as our reference rate, 40 participants per group were required.

Demographic and clinical information were recorded at baseline. Statistical analysis was performed using the Statistical Package for the Social Sciences (SPSS ver. 17.0.1, SPSS Inc., Chicago, IL, USA). Normality of data distribution was assessed using the Kolmogorov-Smirnov test. An unpaired *t* test was performed to compare means of continuous variables that exhibited normal distributions (axial movement, decentration, tilt, visual acuity, refraction and total aberration) between the two groups. Continuous variables without a normal distribution were compared using the Mann-Whitney U test. Categorical variables such as sex were compared using Fisher’s exact probability test. All statistical tests were two-tailed, and a p-value below 0.05 was considered statistically significant. The results are presented as the mean ± standard deviation (SD).

**Chapter 6**

**References**

1. Nagy ZZ, McAlinden C: Femtosecond laser cataract surgery. Eye Vis (Lond) 2015, 2:11.
2. Korynta J, Bok J, Cendelin J: Changes in refraction induced by change in intraocular lens position.J Refract Corneal Surg 1994, 10(5):556-564.
3. Wang L, Koch DD: Effect of decentration of wavefront-corrected intraocular lenses on the higher-order aberrations of the eye. Arch Ophthalmol 2005, 123(9):1226-1230.
4. Rosales P, Marcos S: Phakometry and lens tilt and decentration using a custom-developed Purkinje imaging apparatus: validation and measurements. J Opt Soc Am A Opt Image Sci Vis 2006, 23(3):509-520.
5. de Castro A, Rosales P, Marcos S: Tilt and decentration of intraocular lenses in vivo from Purkinje and Scheimpflug imaging. Validation study. J Cataract Refract Surg 2007, 33(3):418-429.
6. Dolgin E: The myopia boom. Nature 2015, 519(7543):276-278.
7. Schulz KF, Grimes DA: Unequal group sizes in randomised trials: guarding against guessing. Lancet 2002, 359(9310):966-970.
8. Pan CW, Liu H, Sun HP, Xu Y: Increased Difficulties in Managing Stairs in Visually Impaired Older Adults: A Community-Based Survey. PLoS One 2015, 10(11):e0142516.
